# Optimization and Preparation of Ultrasound-Treated Whey Protein Isolate Pickering Emulsions

**DOI:** 10.3390/foods13203252

**Published:** 2024-10-13

**Authors:** Nan Li, Xiaotong Zhang, Juan Zhu, Yinta Li, Rong Liu, Peng Zhang, Suzhen Wei, Xuejun Fu, Xinyan Peng

**Affiliations:** 1 College of Life Science, Yantai University, Yantai 264005, China; linan20010908@163.com (N.L.); zhangxiaotong0622@163.com (X.Z.); zhujuan8223@163.com (J.Z.); fuxuejun@126.com (X.F.); 2 Weihai Key Laboratory of Medical Conditioning Functional Food Processing Technology, Weihai Ocean Vocational College, Weihai 264300, China; liyinta123@163.com (Y.L.); lr870307@163.com (R.L.); wsz3322605@126.com (S.W.); 3 College of Pharm, Yantai University, Yantai 264005, China; zp19877891@126.com

**Keywords:** whey protein isolate, ultrasound, Pickering emulsions

## Abstract

This study aimed to create Pickering emulsions with varying oil fractions and assess the impact of ultrasonic treatment on the properties of Whey Protein Isolates (WPIs). At 640 W for 30 min, ultrasound reduced WPI aggregate size, raised zeta potential, and improved foaming, emulsifying, and water-holding capacities. FTIR analysis showed structural changes, while fluorescence and hydrophobicity increased, indicating tertiary structure alterations. This suggests that sonication efficiently modifies WPI functionality. Under ideal conditions, φ = 80 emulsions were most stable, with no foaming or phase separation. Laser scanning revealed well-organized emulsions at φ = 80. This study provides a reference for modifying and utilizing WPI.

## 1. Introduction

Due to its nutritional, functional, and active qualities, whey—a byproduct of the cheese-making process—can be ultrafiltered to produce whey protein isolate (WPI), a widely used food additive [1]. It is mostly composed of proteins, including β-lactoglobulin (β-LG) and α-lactalbumin (α-LA), with trace amounts of peptone (PP), cryoglobulins (GMP), immunoglobulins (Igs), lactoferrin (LF), lactoperoxidase (LP), and bovine serum albumin (BSA). It is essential to denature and/or unfold spherical dense structures, which are primarily composed of albumin and globulin fractions (ionic groups, hydrophobic groups, and -SH groups), to increase the accessibility of the reactive groups [2].

Because of its cavitation, mechanical, and thermal effects, ultrasound is a commonly utilized technology in the physical preparation of food products [3]. Numerous investigations have demonstrated that sonication is a useful technique for altering proteins to improve their functional characteristics [3]. The phenomena of acoustic cavitation, in which cavitation bubbles form quickly and collapse violently during sonication, may be connected to the effect of ultrasound on liquid systems [4]. Because of the cavitation effect and shear stress from the ultrasonic pretreatment, there are more reactive sites of proteins in protein solutions, which increases the rate of cross-linking reactions [5]. It is commonly known that after ultrasonic pretreatment, proteins release a significant amount of free radicals. Furthermore, it has been documented that free radicals can alter the structural integrity and oxidatively degrade proteins [6].

Pickering emulsions have attracted a lot of attention recently in several areas, such as interfacial catalysis, composites, food, medicine, and cosmetics [7]. A Pickering emulsion is a stable emulsion formed at the interface between two immiscible liquids by the adsorption of colloidal particles [8]. By stabilizing the emulsion droplets, these particles confine oil inside a layer of solid particles [9]. They mainly provide a spatial barrier at the oil–water interface that keeps droplets from coalescing and improves the stability of the emulsion [10,11]. Pickering emulsions have several advantages, including stability, low toxicity, biodegradability, anti-caking properties, and lack of surfactants. However, most investigations have concentrated on inorganic or synthetic polymer particles rather than edible ones [12]. Because of safety and environmental considerations, inorganic particles are not appropriate for use in the food or pharmaceutical industries. The medical utility of natural products has received increased attention due to their comparable safety compared to chemical drugs [13]. Therefore, it is essential to develop natural, environmentally benign, and biodegradable food-grade particles to serve as stabilizers for Pickering emulsions. Thus, this work set out to find out how sonication affects WPIs’ structural and functional characteristics and investigate how various oil-phase volume fractions affect Pickering emulsion preparation. This work will serve as a resource for modifying WPIs to prepare Pickering emulsions.

## 2. Materials and Methods

### 2.1. Materials

WPIs (purity ≥ 95%) were provided by Hilmar Cheese Company (Hilmar, CA, USA) and soybean oil was sourced from Yihai Kerry Arawana Holdings Co., Ltd. (Shanghai, China). The other reagents used in the experiments were of analytical grade.

### 2.2. Preparation of Protein Samples

By dissolving WPI powder in deionized water, a 100 g/L concentration of WPI solution was created. For two hours, the WPI solution was agitated at room temperature. With 0.1 mol/L sodium hydroxide, the pH of the whey isolate protein solution was brought to 7. It was then kept at 4 °C for an entire night until it was dissolved. Ultimately, a KQ-800DE ultrasonic processor (Kunshan Ultrasonic Instrument Co., Ltd., Kunshan, China) processed the WPI solution for 0, 10, 20, 30, 40, 50, and 60 min at an ultrasonic frequency of 40 kHz and ultrasonic powers of 320, 400, 480, 560, 640, 720, and 800 W. The entire ultrasonic treatment was carried out at a regulated temperature of 25 ± 5 °C in an ice–water bath. A 0 min sonication (untreated WPI) was used as a control.

### 2.3. Determination of Particle Size and Turbidity

With a few minor adjustments, particle size was determined in accordance with Sui et al. [14]. A Bettersize 2000 laser particle size distribution meter (Bettersize Instruments Ltd., Dandong, China) was used to ascertain the materials’ particle size distribution. For every sample, three measurements were obtained.

Using the Beer–Lambert law-based spectrophotometric approach developed by Malik et al. [15], turbidity was measured. Deionized water was used to dilute the samples fifty times. The turbidity of the sample solution was determined by measuring its absorbance at 633 nm. Water that had been deionized served as the reference solution.

### 2.4. Determination of Zeta Potentials

The WPI solution was diluted with deionized water at a ratio of 1:50, following the method of Shi et al. [16], with a few minor adjustments. The zeta potential was then measured using a NanoBrook 90Plus Zeta (Brookhaven Instruments, New York, NY, USA). The samples took 60 s to acclimate to 25 °C. After three measurements of each sample, the average value was determined.

### 2.5. Fluorescence Spectra of WPI

An RF-6000 fluorescence spectrophotometer (Shimadzu, Kyoto, Japan) was used to measure the fluorescence emission spectra of the WPI solutions. The WPI solution’s fluorescence spectra were measured between 290 and 450 nm for emission and at 280 nm for excitation. Deionized water was used to dilute the solution concentration to 0.1 mg/mL. Three measurements of each sample were made and the average was recorded.

### 2.6. Determination of Water-Holding Capacity (WHC)

In 50 mL centrifuge tubes, protein samples were weighed and combined. After centrifuging the samples for 10 min at 10,000 g, the supernatant was disposed of. After centrifuging the tube, the centrifuge tube and residue were weighed. This was repeated three times for each size. The following formula can be used to determine a protein sample’s WHC:

WHC (g H_2_O/g protein) = (M_1_ − M_2_)/M_0_ (1)

where M_1_ represents the weight before centrifugation (g), M_2_ represents the weight of the tube and residue (g) after centrifugation, and M_0_ represents the weight of the protein sample (g).

### 2.7. Determination of Surface Hydrophobicity

The surface hydrophobicity (H_0_) of the WPI was determined using the Heldt et al. [17] approach, with some modifications. Initially, 0.01 mg/mL of pH 7.0 phosphate buffer was added to the protein sample solution to dilute it to 0.1, 0.2, 0.3, 0.4, 0.5, 1, 2.5, 5, and 10 mg/mL. Subsequently, each combination was incubated for two minutes at room temperature using 20 μL of 8.0 mmol/L 8-Anilino-1-naphthalenesulfonic acid (ANS) solution (0.01 mol/L phosphate buffer, pH 7.0). The protein samples’ fluorescence intensity was ultimately assessed using an F-5000 UV spectrophotometer (Shimadzu, Kyoto, Japan). The slit between the excitation and emission wavelengths was set to 5 nm and the excitation and emission wavelengths were set at 370 and 484 nm, respectively. It was stated which way the surface hydrophobicity first sloped. Least-squares linear regression analysis was used to calculate this.

### 2.8. Determination of Foaming and Emulsifying Properties

The samples’ foam expansion (FE) and foam stability (FS) were tested using a slightly modified version of Sui et al.’s [14] methodology. To be more precise, 10 mL of the sample was put into a 50 mL plastic centrifuge tube and homogenized for 60 s at room temperature using a high-speed homogenizer (Ultra-Turrax T18, IKA, Staufen, Germany) at 12,000 rpm. To obtain the FE, the entire volume after mixing was measured right away. The samples were allowed to rest for 30 min and the total volume was measured to compute the FS. The equations for the calculation of FE and FS were as follows:

FE(%) = V_T_/V_0_ × 100 (2)

FS(%) = V_t_/V_0_ × 100 (3)

where V_0_ is the initial volume before high-speed mixing, V_t_ is the total volume following 30 min of standing, and V_T_ is the total volume following high-speed mixing.

The emulsion activity (EAI) and emulsion stability (ESI) were determined by referring to the method of Peng et al. [18], with necessary adjustments. First, a high-speed homogenizer (Ultra-Turrax T18, IKA, Staufen, Germany) was used to combine 1 mL of soybean oil with 3 mL of the WPI sample. This emulsion was run for one minute at 10,000 rpm. At 0 and 30 min, 50 μL was taken out of the bottle’s bottom and added to 5 mL of 0.1% (*w*/*v*) sodium dodecyl sulfate (SDS) solution. At 500 nm, absorbance was measured. As a control, a 0.1% SDS solution was employed. The following are the expressions for the emulsion stability index (ESI) and emulsifying ability index (EAI):

(4) $$EAI(m^{2}/g)=\frac{2\times 2.303}{C\times (1-\varphi )\times 10}\times A\times D$$

(5) $$ESI(\%)=\frac{A_{30}}{A}\times 100\%$$

where C is the sample concentration (g/mL) 0.1, V is the volume fraction of the oil phase (V = 1/4), D is the dilution factor, A_30_ is the absorbance of the emulsion absorbed for 30 min, and A is the absorbance of the emulsion absorbed for 0 min.

### 2.9. Fourier Transform Infrared Spectroscopy (FTIR) of WPI

Following sonication, FTIR spectroscopy was employed to track modifications in the WPIs’ secondary structure. A Lab-1A-50 vacuum freeze-dryer (BoYiKang instrument Co., Ltd., Beijing, China) was used to freeze-dry the samples. An FTIR spectrophotometer (Perkin-Elmer, Shelton, CT, USA) was used to measure the FTIR spectra, with a resolution of 4 cm^−1^ from 400 cm^−1^ to 4000 cm^−1^. Fourier transform infrared spectra were recorded.

### 2.10. Preparation of Pickering Emulsion

An Ultra-Turra T18 (IKA, Staufen, Germany) high-speed shear disperser homogenized the modified whey isolate protein solution (640 W ultrasonication for 30 min at 100 g/L) and the soybean oil to a total volume of 40 mL. The modified whey isolate protein solution had a mass concentration of 100 mg/mL. The volume fraction of the oil phase was adjusted to 30%, 40%, 50%, 60%, 70%, and 80% (φ = 30, 40, 50, 60, 70, and 80). To create a stable Pickering emulsion, homogenization was performed using the high-speed shear disperser Ultra-Turra T18 (Ultra-Turrax T18, IKA, Staufen, Germany). High-speed shear was used at 12,000 rpm for three minutes.

### 2.11. Stability of Pickering Emulsions

#### 2.11.1. Storage Stability of Pickering Emulsions

Newly made Pickering emulsions were put into a ten-milliliter centrifuge tube and left to stand at 4 and 25 °C for thirty days to see how the emulsion looked. On days 0, 10, 20, and 30, the centrifuge tubes were photographed to look for any alterations, including layering or separation.

#### 2.11.2. Thermal Stability of Pickering Emulsions

Heat treatment was used to confirm the WPI Pickering emulsions’ thermal stability. The emulsions were heated in a water bath for 30 min at 50 °C, 60 °C, 70 °C, and 80 °C. To evaluate the emulsions’ thermal stability, their appearance was photographed and documented.

#### 2.11.3. Centrifugal Stability of Pickering Emulsions

The assessment of centrifugal stability involved comparing the outside appearance of freshly made emulsions before and after centrifugation at 4000× *g* for 10 min.

### 2.12. Confocal Laser Scanning Microscopy (CLSM) of Pickering Emulsions

Using a confocal laser scanning microscope (LSM 980, Carl Zeiss Company, Oberkochen, Germany), the Pickering emulsion’s morphology was assessed by Zhang, Liu, et al.’s methodology [19]. In summary, 30 min was spent incubating 1.0 mL of freshly made emulsion in the dark; 20 μL of Nile blue solution (1 mg/mL) was used for protein staining and 25 μL of Nile red solution (1 mg/mL) was used for oil staining. Excitation wavelengths of 633 nm for Nile Blue and 488 nm for Nile Red were used to record observations.

### 2.13. Statistical Analysis

Utilizing statistical analytic techniques and analysis of variance, the different treatments were evaluated referring to the Pickering emulsion quality criteria. Statistix (version 8.1 Analytical Software International, Tallahassee, FL, USA) software was used in all statistical analyses. We utilized Tukey’s multiple comparisons in conjunction with analysis of variance (ANOVA) to ascertain the significance of treatment effects (*p* < 0.05). Every one of the three experiments had three duplicates. The data are shown in the format mean ± standard deviation.

## 3. Results and Discussion

### 3.1. Particle Size and Turbidity

**Figure 1:**
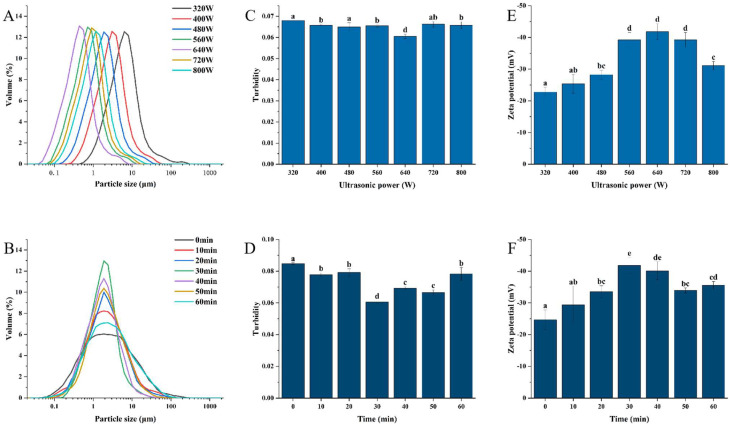
Changes in particle size (**A**,**B**), turbidity (**C**,**D**), and zeta potential (**E**,**F**) of samples after sonication. (**A**,**C**,**E**) denote the experimental results under different treatment powers; (**B**,**D**,**F**) denote the experimental results under different treatment times. Data are presented as mean ± SD. Different letters indicate significant differences (*p* < 0.05).

The functional characteristics of proteins are intimately associated with the particle size in protein solutions [20]. Figure 1A,B displays the particle size distributions of WPIs at various power (320, 400, 480, 560, 640, 720, 800 W) and time (0, 10, 20, 30, 40, 50, 60 min) conditions of ultrasonication. All samples had symmetric, narrow, unimodal apparent size distributions. With a peak of around 1.885 μm, the reduction in particle size was most noticeable after 30 min of 640 W ultrasonic treatment. Similar to this, Gammoh et al. showed how ultrasonication can change the distribution of casein and whey proteins in camel milk by increasing the quantity of medium-sized particles, which causes the z mean diameter to be halved [21]. Hu et al.’s [22] study examined how the particle size distribution of soybean isolate protein particles was affected by applying high-intensity ultrasound for 5, 20, or 40 min. Additionally, they noticed that during the first 20 min, the volume–mean diameter (D43) of the soy protein dispersions significantly decreased. The main mechanisms involved were ultrasonic cavitation, microfluidics, and turbulent forces, which reduced the size of macromolecules, disrupted protein aggregation, increased the surface area of the particles, and improved inter-particle interactions. It has been reported that ultrasonic treatment significantly affects the particle size reduction of various protein suspensions, including whey proteins [23]. After ultrasonic treatment, the energy required to hydrolyze the peptide bonds is insufficient, leading to a significant change in the structure and a decrease in particle size. This disruption of electrostatic interactions includes hydrogen bonds, van der Waals forces, and the partial cleaving of intermolecular hydrophobic interactions instead of peptide or disulfide bonds [24]. Then, an increase in particle size can be noticed, which might be the result of protein molecule agglomeration and thermal effects in the system from high power and extended sonication, where a temperature rise could cause particles to agglomerate [25].

Turbidity is utilized to quantify the extent of protein aggregation and serves as a reflecting indicator of colloidal and suspended particles in WPI solutions [26]. Figure 1C,D displays the turbidity of WPI that was sonicated and left untreated. As the ultrasonic power was increased from 320 to 800 W and the ultrasonic time was increased from 0 to 60 min, the turbidity of WPI suspensions decreased and then increased with the change in particle size. The turbidity of WPI was significantly reduced at 30 min of ultrasonic power of 640 W (*p* < 0.05). Additionally, Malik et al. showed that ultrasonication [15] and particle size were responsible for a sunflower protein solution’s decreased turbidity. According to Yao et al.’s research [27], the turbidity of WPI suspensions dramatically dropped after 20 to 40 min of ultrasonic treatment and only marginally increased when the ultrasonic power was raised from 200 to 800 W. In addition, the turbidity of WPI suspensions dramatically decreased (*p* < 0.05) as the ultrasonic power was raised even more. Furthermore, the turbidity of WPI increased as a result of protein aggregation, with a further increase in ultrasonic power (>640 W) for a longer duration (>30 min).

### 3.2. Zeta Potentials

The surface charge of particles influences their solubility and interactions with other particles, and zeta potential is a significant characteristic [28]. Protein dispersion and aggregation are impacted by the Zeta potential of particles in solution, which is proportional to their surface or near-surface charge. Stronger electrostatic repulsion between protein particles is indicated by higher absolute values of Zeta potential, which keeps proteins from aggregating and maintains system stability [29]. Charged groups in the WPI are exposed as a result of the collapse and rupture of cavitation bubbles created during ultrasonic treatment, which raises the surface charge number and the zeta potential’s absolute value [30]. The ionization of surface groups is the primary source of a protein’s surface charge. The reduction in surface charge is explained by the exposure of non-polar hydrophobic residues, which may be brought about by the unfolding of proteins that have been sonicated [31]. The final surface charge of most proteins is determined by the equilibrium between their hydrophilic polar groups (e.g., hydrophilic polar groups like -OH and -NH_2_), ionic groups like -NH_3_ and -COO- plasma groups, and non-polar hydrophobic residues like aromatic and alkyl groups [32]. Figure 1E,F illustrates the zeta potential of the untreated WPI, which is roughly −22.76 mV. The WPI solution was stabilized after sonication. After 30 min of ultrasound treatment at 640 W, the highest absolute value of the sample potential (*p* < 0.05) was detected compared to untreated WPI. The proteins re-aggregated when the ultrasonic power was increased (>640 W) and for a longer duration (>30 min), burying the previously exposed polar locations [33]. The potential’s absolute value decreased as a result of a drop in surface charge. Particle stability and interactions with other particles were both influenced by their surface charge [34]. One of the key variables influencing the foaming capabilities of proteins has been observed to be the value of the protein’s zeta potential [33]. The qualities of protein foam are generally greater the higher the absolute value of zeta potential.

### 3.3. Fluorescence Spectra

**Figure 2:**
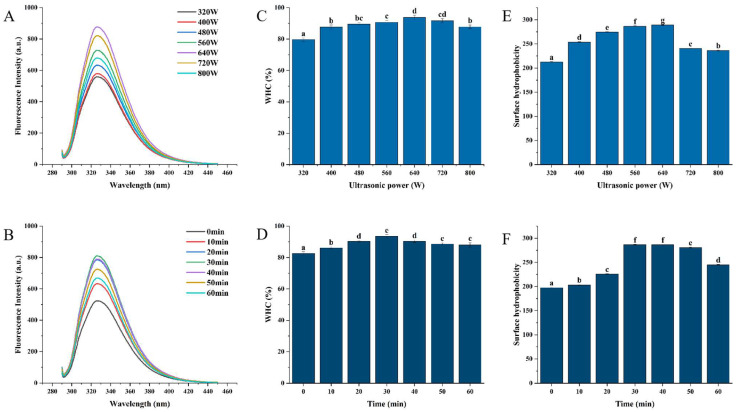
Changes in fluorescence intensity (**A**,**B**), water-holding capacity (**C**,**D**), and surface hydrophobicity (**E**,**F**) of samples after ultrasonic treatment. (**A**,**C**,**E**) denote the experimental results under different treatment powers; (**B**,**D**,**F**) denote the experimental results under different treatment times. Data are presented as mean ± SD. Different letters indicate significant differences (*p* < 0.05).

The samples’ fluorescence spectra were taken to better examine how non-covalent interactions and sonication affect the polar and structural alterations of WPI. Tryptophan, phenylalanine, and tyrosine residues in proteins—especially the latter—have extremely sensitive fluorescence intensities that are affected by the polarity of a solvent. Conformational changes in WPI are correlated with either an amplification or a quenching of these residues’ fluorescence. Consequently, the goal of intrinsic fluorescence monitoring is to spot modifications in the tertiary structure of proteins that are associated with extrinsic factor interference and the creation of complex conjugates [35]. The fluorescence variations of WPI are depicted in Figure 2A,B which first exhibited a growing trend before declining as the ultrasonic power and time increased. Strong cavitation caused the increasing exposure of tryptophan residues that were previously hidden in the WPI structure as a result of sonication, which was the cause of this trend [30]. This aligns with the research conducted by Zhao et al. [21] and Yan et al. [36]. On the other hand, a minor decrease in fluorescence intensity was noted with additional increases in sonication power and duration. This implies that some of the exposed chromophores may have been reburied under excessively high or prolonged ultrasonic power because of changes in protein structure that led to large protein clumps and decreased fluorescence intensity.

### 3.4. Water-Holding Capacity

Water-holding capacity (WHC) and sensory perception are linked, as is the release of fat and water [37]. Figure 2C,D illustrates how, after 30 min, the WHC of WPI grew and then reduced as the ultrasonic treatment’s strength increased. When compared to untreated WPI, the WHC increased by 4.81%, 9.35%, 13.23%, 9.39%, 7.23%, and 6.58% (*p* < 0.05) at 640 W. The surface hydrophobicity and free sulfhydryl groups generated by cavitation were enhanced by sonication. This promoted the creation of disulfide bonds between the exposed free sulfhydryl groups and the development of protein–protein interactions during heating [38]. Proteins expand to reveal hydrophobic residues as well as additional residues that form hydrogen bonds with water with ease. Under the influence of covalent and non-covalent bonding, these border water molecules may burrow into a network’s internal interstices while creating a system with a specific three-dimensional network structure [39]. Furthermore, the protein molecules are more pliable due to the structural alteration, which could account for the WHC value of the sonication treatment being higher than that of the control group. In the meantime, WPI’s particle size decreased following sonication, which encouraged the creation of a more uniform, finer solution with a denser network structure and lowered the rate of water loss [40]. When the sonication time exceeded 30 min and the power exceeded 640 W, the samples’ WHC no longer increased. This could have been a result of the WPI often having a high WHC that was challenging to raise. Similarly, Zisu et al. [41] found that WPI had less of an impact than WP concentrate (WPC), which they attributed to the aggregate particle size’s different composition (WPC had a bigger aggregate particle size than WPI).

### 3.5. Surface Hydrophobicity

The synthesis of proteins and their subsequent functional characteristics, such as solubility, gelation, and emulsification, are reflected in surface hydrophobicity [42]. Because proteins have a strong attraction to hydrophobic surfaces, ANS can be used to measure changes in hydrophobic sites and tertiary structures that are exposed to proteins [43]. Figure 2E,F illustrates the surface hydrophobicity of both untreated and sonicated WPI. Following modification, all evaluated samples exhibited a significant (*p* < 0.05) increase in surface hydrophobicity, indicating that the protein’s tertiary structure was impacted by the ultrasonic modification process. This suggests that the proteins’ structures were changed and that it was not possible to return the proteins to their native configuration. The unfolding of globular proteins exposed hydrophobic amino acids and caused some primary proteins to reorganize, increasing the hydrophobicity of their surfaces. This outcome is in keeping with research by Lam and Nickerson [44], who discovered that in extremely acidic or alkaline environments, protein molecules’ spatial conformation might expand and their surface hydrophobicity is increased. Soy protein, peanut protein, and rice protein have all shown comparable outcomes [45]. The ultrasound modification technique had some effect on the tertiary structure of proteins, which was altered, leading to the exposure of hydrophobic amino acids and the rearrangement of some primary proteins, which enhanced the surface hydrophobicity [46]. When 640 W ultrasound was applied to WPI for 30 min, the hydrophobicity of the surface was considerably (*p* < 0.05) higher than that of the untreated WPI. It is possible that the unfolding of protein molecules, which results in more non-covalent bond extension and hides hydrophobic areas, was the cause of WPI’s decreased surface hydrophobicity at higher ultrasonic powers and longer ultrasound durations [47]. High-power ultrasonic treatment was also found to decrease the hydrophobicity of a black bean protein isolate [31].

### 3.6. Foaming and Emulsifying Properties

**Figure 3:**
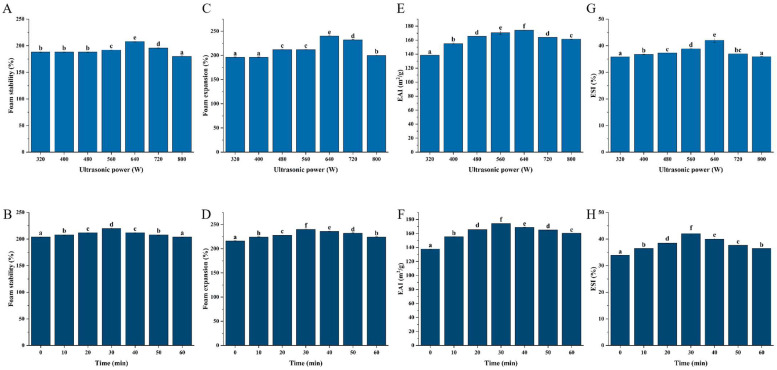
Changes in foam stability FS (**A**,**B**), foam expansion FE (**C**,**D**), emulsion stability ESI (**E**,**F**), and emulsion activity EAI (**G**,**H**). (**A**,**C**,**E**,**G**) denote the experimental results under different treatment powers; (**B**,**D**,**F**,**H**) denote the experimental results under different treatment times. Data are presented as mean ± SD. Different letters indicate significant differences (*p* < 0.05).

Interfacial characteristics are influenced by the various architectures of natural proteins and their aggregation. Foam stability and foaming ability vary as a result [48]. Protein foaming characteristics are associated with their capacity to form films at the gas–liquid interface and may be impacted by their molecular makeup [49]. The foaming characteristics of both sonicated and untreated WPIs are shown in Figure 3A–D. The foaming performance and foam stability first increased and then decreased with an increase in ultrasonic power and an extension of ultrasonic time. The maximum value was reached at 640 W for 30 min, indicating that moderate aggregates were uniformly distributed on the gas–liquid interface. The exposure of hydrophobic groups can somewhat offset the interfacial diffusivity loss brought on by the larger aggregate size. Simultaneously, this exposure improves the stability of protein foaming by facilitating the development of multilayered protein films at the interface [50]. According to Sheng et al., high-intensity ultrasonication reduces the viscosity and surface tension of egg proteins, facilitating easier absorption of the proteins into the gas–liquid interface [51]. This enhances the foaming ability of egg proteins. However, because of their steric hindrance, large-scale protein aggregates are not helpful for adsorption at the interface [52]. According to Ma et al.’s experimental results [53], chickpea protein FS tended to grow and subsequently drop as homogenization pressure increased. This phenomenon could be attributed to the protein molecules becoming denatured under repeated homogenization treatments.

Proteins’ emulsifying qualities, or their capacity to create stable emulsions, are crucial for a variety of food items, including drinks, sauces, and sweets [43]. Protein molecules become more flexible as a result of denaturation and polydispersity caused by ultrasonication, which effectively adsorbs denatured protein molecules at the oil–water interface [54]. The EAI of WPI samples was elevated by ultrasonic treatment (*p* < 0.05, Figure 3E,F. The EAI of ultrasonically treated WPI grew by 21.37%, 46.35%, 72.24%, 56.90%, 40.99%, and 27.38%, respectively, as the duration increased from 0 min to 60 min (*p* < 0.05). Furthermore, it was found that a 30 min exposure to an ultrasonic power range of 640 W considerably improved the emulsion stability of WPI suspensions (*p* < 0.05). This could be connected to specific modifications in surface hydrophobicity and particle size brought on by extended ultrasonic duration [55]. The WPI structure was altered by ultrasonication, which also enhanced the hydrophobic contacts on its surface (Figure 2E,F). In this instance, the emulsification efficiency was increased due to the involvement of additional proteins in the creation of the interfacial membrane [56]. Similarly, it has been demonstrated that ultrasonic treatment increases the amount of small molecule soluble proteins that can connect to the oil–water interface, which, in turn, improves the EAI of protein isolates from walnut and amaranth [47]. According to Wong et al.’s [57] research, the SPI’s functional qualities are enhanced by ultrasonic treatment. This is because it increases the surface hydrophobicity of the particle and reduces its size, both of which enhance emulsification performance. When sonication was applied to WPI, the ESI rose (*p* < 0.05) in comparison to WPI that had not been treated (Figure 3G,H). This could be explained by the sonication-induced production of polymers or oligomers, which stabilized the interfacial membrane [16]. However, the ESI value of the samples dropped when the sonication period exceeded 30 min and the power was above 640 W. This could have been because the samples were over-treated, which led to a significant amount of protein unfolding.

### 3.7. Fourier Transform Infrared Spectroscopy (FTIR)

**Figure 4:**
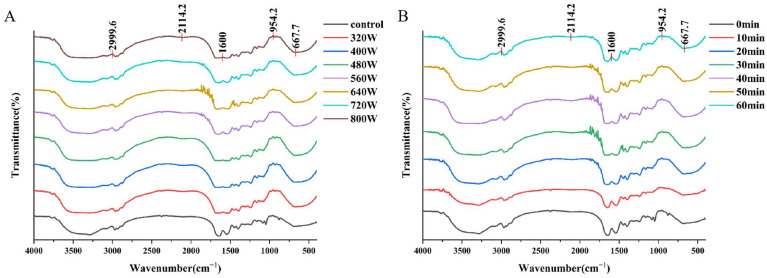
FTIR spectra of untreated and sonicated WPI. Control: WPI samples without any treatment. (**A**) denotes the experimental results under different treatment powers; (**B**) denotes the experimental results under different treatment times.

Protein secondary structures can have their conformational variations identified via the widespread application of Fourier transform infrared (FTIR) spectroscopy. Information regarding the secondary structure of proteins (α-helix, β-folding, etc.) can be obtained from the C-O stretching vibration of the peptide bone in the 1700–1600 cm^−1^ band (amide I band). As per the methodology expounded by Meng et al. [43], the deconvoluted subpeaks can be subsequently categorized to ascertain the secondary structure of WPI. The most helpful part of the IR spectrum for analyzing the secondary structure of proteins is the α-helix, which is centered at 1650–1660 cm^−1^; the β-fold structure, which is centered at 1610–1640 cm^−1^ and 1680–1700 cm^−1^; the β-turns, which are centered at 1660–1680 cm^−1^; and the random curls, which are centered at 1640–1650 cm^−1^. As shown in Figure 4, the stronger absorption peaks at 1600–1700 cm^−1^ of WPI after 30 min of sonication at 640 W indicate that sonication under this condition changed the structure of the protein, which led to a difference in the IR absorption spectra. This may be related to the enhancement of its amide I band, which is usually caused by changes in the secondary structure of the protein, such as a decrease in α-helix and β-fold content. Similarly, after ultrasonication, some researchers have observed changes in the secondary structure of β-lactoglobulin and walnut protein samples. These changes could be caused by the breakage of hydrogen bonds that are necessary for the proteins to maintain their secondary structure [20].

### 3.8. Stability of Pickering Emulsions

**Figure 5:**
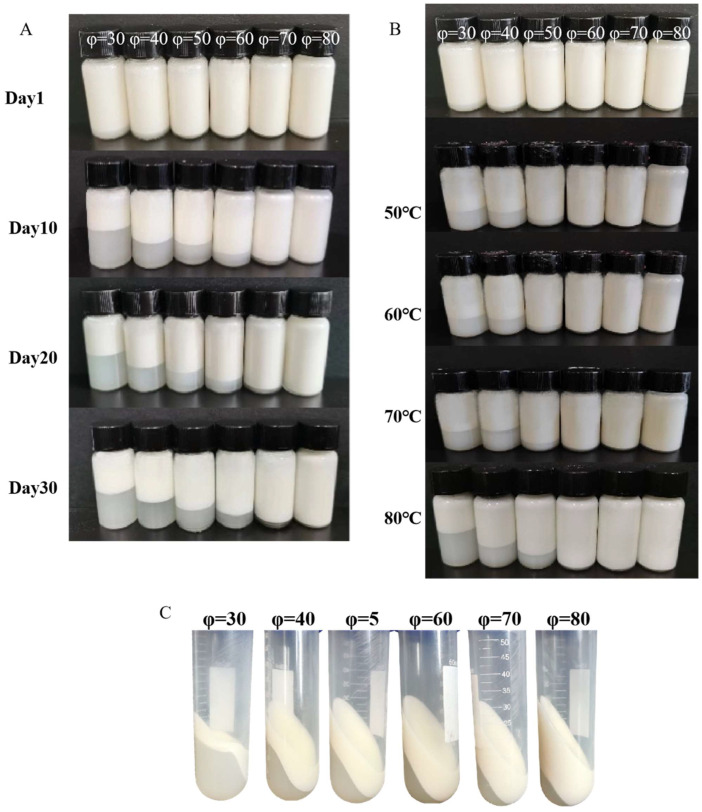
Stability of Pickering emulsions. (**A**) Appearance of Pickering emulsions after 30 days of storage. (**B**) Appearance of Pickering emulsions after heating at 50, 60, 70, and 80 °C, respectively. (**C**) Appearance of Pickering emulsions after centrifugation.

In food matrices, emulsions are utilized to encapsulate and transport bioactive ingredients. The size of oil droplets or the interfacial structure—whether crosslinked or not—has a direct bearing on stability [58]. Emulsions’ storage status can be well described by macroscopic images. The macroscopic pictures of stabilized Pickering emulsions with varying oil phase contents that were kept at 4 °C for 0, 10, 20, and 30 days are displayed in Figure 5A. Except for φ = 80, all emulsions exhibited notable delamination without oil leakage. Under gravity, the water layer of the emulsion is below and the oil-rich emulsion is above. The best physical stability and excellent prevention of protein flocculation are shown by φ = 80. As the volume fraction of the oil phase increased, the Pickering emulsions’ storage stability grew progressively. Pickering emulsions with a smaller volume fraction of the oil phase were more likely to experience emulsion precipitation during storage. This may be ascribed to the fact that the increase in volume fraction of the oil phase increases the viscosity of the emulsion, which slows down the migration of droplets and so enhances the stability of the emulsion.

The pasteurization of food is typically coupled with heat treatment techniques, such as elevated temperatures. Thus, to ascertain the thermal stability of Pickering emulsions, it is necessary to take into account the impact of heating on the emulsions. By examining the morphology of the emulsions at various heating temperature conditions, the thermal stability was evaluated [59]. Following heating treatment, the homogeneity gradually deteriorated and the emulsion delaminated(Figure 5B). This modification had two primary causes. On the one hand, the emulsion traveled violently between droplets following the heating treatment, which lowered the likelihood of droplets meeting and causing oil droplet aggregation. Nevertheless, heating treatment caused the structure of proteins to unfurl and hydrophobic groups to become visible. This encouraged protein molecules to combine through non-covalent interactions, leading to bigger particle sizes [60]. Following centrifugation, which worked effectively under certain centrifugation conditions as the volume fraction of the oil phase grew, a similar pattern became visible(Figure 5C).

### 3.9. Confocal Laser Scanning Microscopy (CLSM)

**Figure 6:**
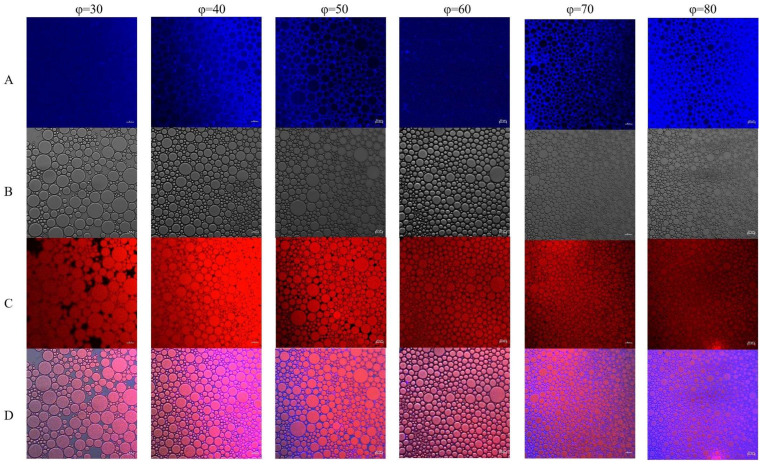
CLSM images of Pickering emulsions with different oil fractions. (**A**) Protein stained with Nile blue. (**B**) Bright field. (**C**) Soybean oil stained with Nile red. (**D**) Overlay image. Scale bar: 20 µm.

The distribution of solid particles at the two-phase interface and the interfacial microstructure of the WPI-stabilized Pickering emulsion were examined using CLSM. The protein particles and oil phase are denoted by the blue and red fluorescence fields, respectively, in Figure 6. All of the emulsions showed a distinct dense particle layer encircling the oil droplets, indicating that they were all O/W. A physical barrier that stopped droplets from accumulating was formed as a result of this thick coating on the droplets’ surface [61]. Particle aggregates proliferated when the oil phase volume percent was less than 0.6, and the droplet size shrank as the oil phase volume fraction rose. There was a dip in droplet size at φ = 80. The majority of the oil droplets had a network structure resembling gel because they were tightly packed together and challenging to move. This strong interfacial structure prevented oil droplets from agglomerating and offered the Pickering emulsion good physical stability. As the oil phase volume percentage rose, the droplet size of EG made with chitosan and high-pressure generated WPI decreased, improving stability [62]. Similar self-assembled network structures were seen by Li et al. [63] in High Internal Phase Emulsions (HIPEs) made with olive oil and carnitine particles, which enhanced stability by preventing droplet aggregation.

**Figure 7:**
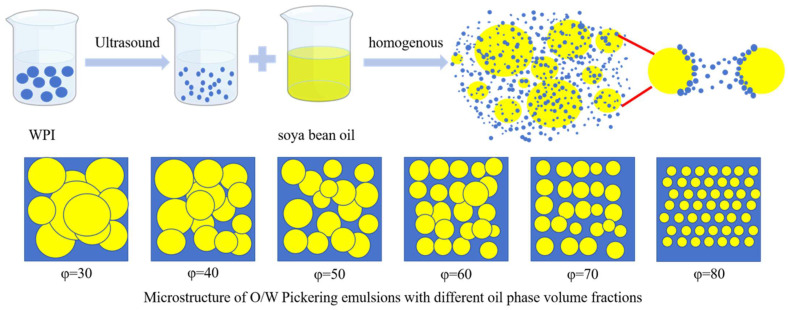
Schematic flow diagram for the preparation of Pickering emulsions.

As shown in Figure 7, in the present study, the effect of treatment with ultrasonic conditions on WPI was investigated in depth. Subsequently, the feasibility and stability of the preparation of Pickering emulsions with the addition of different volume fractions of the oil phase was investigated. The experimental results show that ultrasonic treatment can significantly alter the structure and properties of whey isolate proteins, affecting their role in emulsion formation and stabilization.

## 4. Conclusions

The WPI samples’ aggregate size and turbidity were greatly decreased, and 30 min of 640 W ultrasonication enhanced the zeta potential. Furthermore, the WPI samples’ foaminess, emulsification, and water-holding ability were enhanced with the use of ultrasonication. The secondary and tertiary structures of WPI were changed by ultrasonication, resulting in notable increases in the amount of β-turn and random curl, fluorescence intensity, and surface hydrophobicity. A significant factor affecting the stability of Pickering emulsions stabilized by sonicated WPI was the volume percentage of the oil phase. A more compact structure gradually evolved when the oil volume percentage increased from 30% to 80%. This resulted in a decrease in droplet spacing. Pickering emulsions with an oil volume percentage of 80% demonstrated superior centrifugal, thermal, and storage stability. This study not only shows how ultrasonic treatment can enhance the functional qualities of WPI but also offers a quick and easy way to make WPI-based Pickering emulsions, which can be used to increase the use of WPI as a functional food ingredient and increase its potential in food applications.

## Data Availability

The original contributions presented in the study are included in the article, further inquiries can be directed to the corresponding author.
